# Posterior epistaxis management: review of the literature and proposed guidelines of the hellenic rhinological-facial plastic surgery society

**DOI:** 10.1007/s00405-023-08310-4

**Published:** 2023-11-30

**Authors:** Ioannis Koskinas, Timoleon Terzis, Christos Georgalas, Georgios Chatzikas, Georgios Moireas, Aristidis Chrysovergis, Stefanos Triaridis, Jannis Constantinidis, Petros Karkos

**Affiliations:** 1 1st Academic Otolaryngology Department, AHEPA University Hospital, Aristotle University of Thessaloniki, Kiriakidi 1 Str, 546 21 Thessaloniki, Greece; 2 Hellenic Rhinological-Facial Plastic Surgery Society, Thessaloniki, Greece

**Keywords:** Posterior epistaxis, Management, Guidelines, Early SPA ligation, Nasal packing, Embolization

## Abstract

**Purpose:**

Posterior epistaxis is a common emergency in ENT practice varying in severity and treatment. Many management guidelines have been proposed, all of which are a product of retrospective analyses due to the nature of this pathology, as large-scale double-blind studies are impossible—even unethical—to conduct. The purpose of this review is to perform a thorough analysis and comparison of every treatment plan available and establish guidelines for the best possible outcome in accordance to every parameter studied. Given the extensive heterogeneity of information and the multitude of studies on this topic, along with the comparison of various treatment options, we opted for a literature review as our research approach.

**Methods:**

A review of the literature was performed using PubMed Database and search terms included “posterior epistaxis”, “treatment”, “management”, “guidelines”, “algorithm” “nasal packing”, “posterior packing”, “surgery”, “SPA ligation”, “embolization”, “risk factors” or a combination of the above.

**Results:**

Initial patients’ assessment invariably results in most cases in posterior packing. There seems to be a superiority in recent literature of early surgery over nasal packing as a definitive treatment. Embolization is usually used after surgery failure, except for specific occasions.

**Conclusion:**

Despite the vast heterogeneity of information, there seems to be a need for re-evaluation of the well-established treatment plans according to more recent studies.

**Graphical abstract:**

**Figure:**
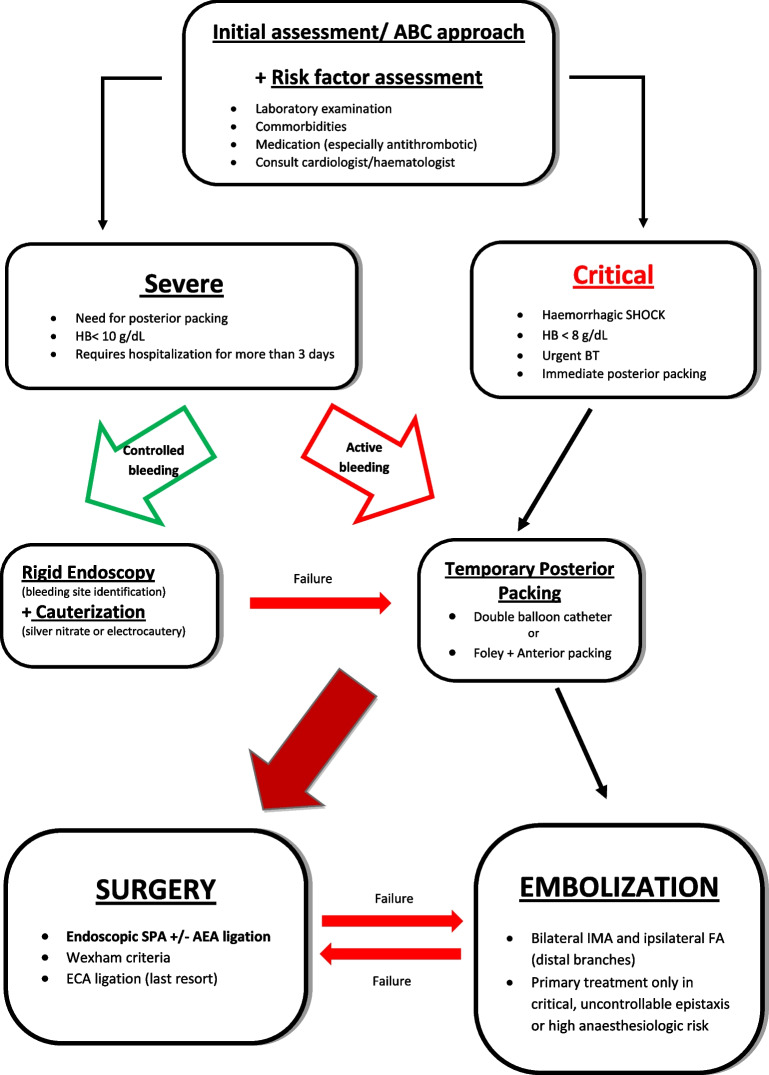
Suggested treatment algorithm for posterior epistaxis. *ABC* Airway-Breathing-Circulation, *ECA* external carotid artery, *AEA* anterior ethmoidal artery, *BT* blood transfusion, *FA* facial artery, *HB* haemoglobin, *IMA* internal maxillary artery, *SPA* sphenopalatine artery

## Introduction

Posterior epistaxis, a serious form of nosebleeds, mainly affects the late adulthood (mostly the fifth or sixth decades). At least 60% of the general population encounters an episode of epistaxis during their lifetime, out of which approximately 6% of cases encounter severe and intractable bleeding, requiring immediate medical assistance, management, and hospitalization [1, 2]. It is estimated that severe epistaxis constitutes 30% of all emergency otolaryngology cases, highlighting the need to establish contemporary guidelines for its favorable management [22]. Numerous guidelines have been proposed in the past, primarily deriving from retrospective analyses due to the inherent characteristics of this medical condition. The purpose of this review is to perform a thorough analysis and comparison of every treatment plan available and establish guidelines for the best possible outcome in accordance to every parameter studied. Considering the substantial diversity of information available and the abundance of studies on this subject, as well as the necessity to compare different treatment options, we have chosen to employ a literature review as our research methodology. Posterior epistaxis evaluation and treatment focus on parameters, such as bleeding origin, severity, recurrence, predisposing factors, and logistics.

## Materials and methods

### Study design

This comprehensive literature review was designed to provide an overview of the current opinions and management strategies for posterior epistaxis, with a focus on recent literature. The primary aim was to propose evidence-based and straightforward guidelines for optimal management. The research question was formulated using the PICOT structure: In adults with posterior epistaxis (P), what are the reported outcomes of various treatment strategies (I), compared to other methods or standard care (C), in terms of efficacy, safety, and patient satisfaction (O) in literature published after 2005 (T)? The primary aim of this review was not to conduct a systematic review, due to the broad and complex nature of the topic, but to provide solid arguments regarding the current opinions on posterior epistaxis management. Furthermore, this article aims to elucidate the application of antibiotics and provide guidance on the administration of antithrombotic medication, the latter of which often necessitates a collaborative, multidisciplinary approach.

### Database and search terms

A review of the literature was performed using PubMed Database and search terms included “posterior epistaxis”, “treatment”, “management”, “guidelines”, “algorithm” “nasal packing”, “posterior packing”, “surgery”, “SPA ligation”, “embolization”, “risk factors”, or a combination of the above.

### Inclusion criteria

“The studies included in this review met the following criteria:

1. Published in the English language.
2. Case series with more than 10 patients.
3. Prospective and retrospective studies.
4. Systematic reviews and meta-analyses.
5. Literature published after the year 2005.”

### Exclusion criteria

“Studies were excluded from this review if they were:

1. Single case reports, except for a single case report that was included due to its clinical significance and rarity.
2. Case series with fewer than 10 patients.
3. Non-English literature.
4. Literature published before the year 2005.”

### Selection process

A total of 394 articles were initially identified through literature search, all published after the year 2005. Those fitting the inclusion criteria were selected for full-text review. Case reports, case series with fewer than ten patients, non-English literature, and irrelevant articles were excluded from this review. The titles and abstracts of the articles chosen from the database search were reviewed. All articles chosen were published after the year 2005 and included case series with more than ten patients, case–control studies, cohort studies, retrospective studies, systematic reviews, and meta-analyses. This review does not strictly adhere to a systematic review approach due to the broad and complex nature of the topic. However, all articles were thoroughly reviewed and assessed by the Hellenic Rhinological-Facial Plastic Surgery Society, providing a robust analysis based on expert opinions and comprehensive analysis of the chosen articles.

### Outcome

The outcomes of this review offer a comprehensive understanding of the current opinions on posterior epistaxis management. The guidelines proposed are derived from a meticulous literature analysis and expert opinions. These guidelines aim to provide an evidence-based and straightforward approach for the optimal management of posterior epistaxis.

## Results

### Definition of posterior epistaxis and key bleeding areas

Posterior epistaxis is generally defined by the inability to visually identify a bleeding point on anterior rhinoscopy using a headlight, due to its origin in a deep crevice of the lateral nasal wall or the posterior part of the septum [1, 4, 5]. Supriya et al. used piriform aperture as an anatomic landmark to divide epistaxis in anterior or posterior [2]. In the absence of a symptomatic tumor, posterior epistaxis can subsequently be divided in lateral nasal wall bleeding, septal bleeding, or nasal floor bleeding [4], the majority of which (80%) originate from the lateral nasal wall and, specifically, from the posterior end of the lateral aspect of the middle and inferior turbinates and the lateral wall of the meati [5]. Woodruff’s plexus, another common bleeding site, is located on the posterior aspect of the lateral wall of the inferior meatus. Other key areas involve the middle and the posterior part of the septum and the floor of the nose beneath the inferior turbinate [1].

### Grades of posterior epistaxis (Fig. 1)

**Fig. 1:**
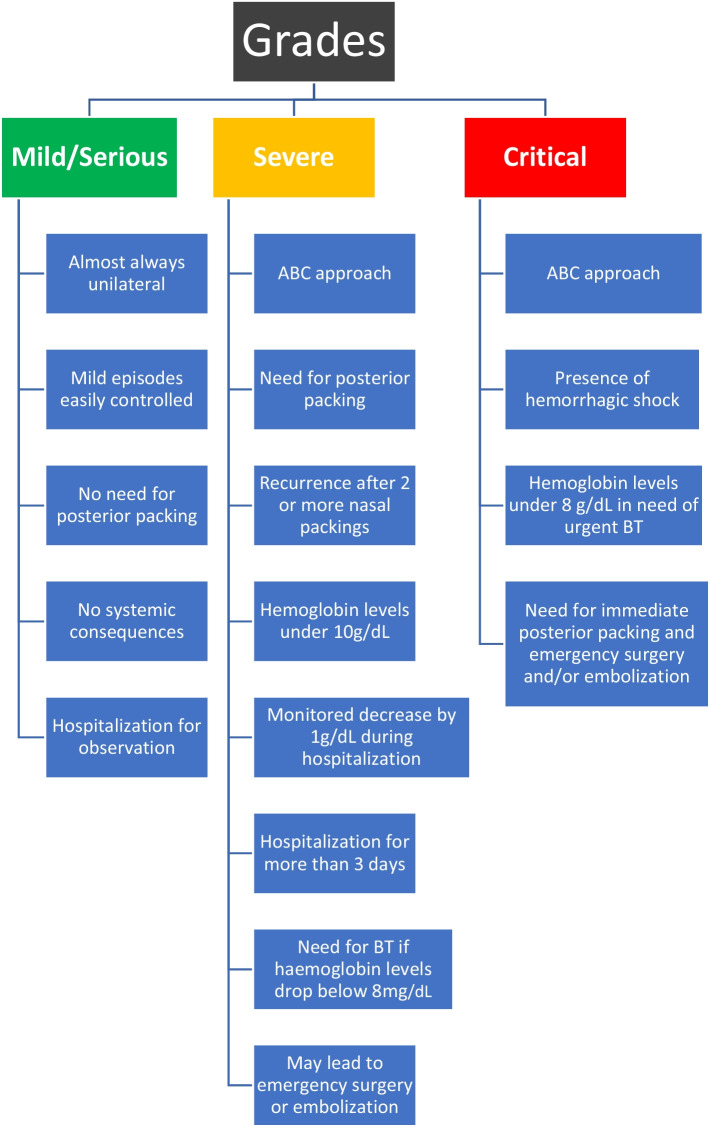
Grades of Posterior Epistaxis According to its Severity

1. Posterior epistaxis is defined as mild or serious if a patient is in the need for medical evaluation and potential intervention [16]. There may be mild blood flow without systemic consequences or decrease in hemoglobin levels [26].
2. Severe epistaxis is defined as more aggressive and in need of posterior packing and hospitalization for more than 3 days. During admission, hemoglobin levels measure under 10 g/dL, without the need for blood transfusion (ΒΤ). The patient’s vital signs remain constant, but a decrease in hemoglobin levels may require ΒΤ if levels drop below 8 g/dL [4]. The patient’s packing is observed for potential recurrence. All these factors may lead to emergency surgery or embolization [16, 23, 24, 26].
3. More frequent and severe bleeding episodes, as well as increased risk for recurrence, are associated with antithrombotic therapy, especially anticoagulant drugs [18, 28].
4. An episode is considered critical when hemorrhagic shock is induced and a drop in hemoglobin levels below 8 g/dL requires immediate ΒΤ [4, 26]. It is imperative to immediately stop or at least slow down the blood loss with a temporary posterior packing, stabilize the patient, and proceed to the treatment [3].

### Predisposing factors

Epistaxis can be classified according to its etiology into primary or secondary. Primary epistaxis occurs in the absence of a specific cause, whereas secondary epistaxis is caused by a definite condition. A specific cause of epistaxis is found in 15% of patients seeking medical assistance, whereas 85% of them receive treatment for a primary idiopathic episode associated with certain risk factors [12]. The risk factors for recurrent epistaxis include congestive heart failure, diabetes, and the use of anticoagulant medications [18].

#### Primary epistaxis

It is important to associate primary epistaxis with predisposing factors, which can potentially lead to a higher bleeding risk. These include age, sex, hypertension, atherosclerosis, environmental causes, and circadian rhythm. Further information is shown in Table 1 [2, 11–14, 28].

**Table 1:** Predisposing factors and etiology of posterior epistaxis

Primary		Secondary	
Age	45–65 years (mean 53 years) Mucosal dryness Decreased elasticity of nasal mucosa No correlation to bleeding site	Hematological	Liver or renal disease Leukemia Myelodysplasia Thrombocytopenia Hemophilia A and B Von Willebrand disease
Sex	Male predominance up to 49 years, after that same frequency between men and women	Neoplastic	Benign (e.g., Juvenile nasopharyngeal angiofibroma) Malignant (Nasal and paranasal tumors) Other lesions (hemangioma)
Hypertension	Possibly due to atherosclerosis	Traumatic	Digital manipulation Iatrogenic (nasal surgery, intranasal interventions) Nasal fracture/ facial trauma Foreign body
Atherosclerosis	Hyperlipidemia Rupture of vessel due to loss of elasticity	Structural	Septal deviation or perforation Hereditary Hemorrhagic Telangiectasia (Osler–Weber–Rendu disease)
Environmental	Mucosal dryness (humidity, temperature, and altitude) CPAP usage	Drug related	Antithrombotic therapy (antiplatelet, anticoagulants and NOACs) Anti-inflammatory drugs Steroid nasal sprays Nasal drug consumption (cocaine) Alternative medicine (e.g., fish oil) Alcohol
Circadian rhythm	Mostly between 4 and 8 am	Inflammatory	Rhinosinusitis Allergy Other infections

##### The role of hypertension and atherosclerosis

Although the prevalence of hypertension in primary epistaxis ranges from 24 to 64%, their cause-and-effect relationship remains a subject of ongoing controversy and uncertainty [15, 16]. Patients tend to have a high measurement upon examination, which is potentially anxiety-related [16]. Uncontrolled hypertension is associated with a higher incidence of epistaxis, possibly as a result of its long-lasting effects in target-organs [12, 15]. Elwany et al. revealed a strong connection between Low-Density-Lipoprotein (LDL) levels and primary epistaxis episodes, as well as recurrent. They found out that hypertensive retinopathy caused by atherosclerosis may be an indicator of nasal vessel damage. Thus, an ophthalmologic assessment could be auxiliary in treating an underlying condition [19]. Severe epistaxis may reveal underlying elevated blood pressure levels in 43% of the patients, and therefore cardiovascular assessment is mandatory [17]. All these findings show that elevated blood pressure may be the result and not the cause of epistaxis, although it may be the cause of recurrence [18]. Oral medication should be administered to reduce blood pressure levels over a span of 24–48 h, resulting in suspension of bleeding in 65–75% of the cases [3].

Kunz et al. argued that patients with so far subclinical atherosclerosis presenting with epistaxis are at greater risk for cardiovascular disease (CVD), and thus, they suggested using the carotid artery intima–media thickness (CIMT) test in epistaxis patients as an indicator for subclinical CVD [20].

#### Secondary epistaxis

A cause of epistaxis is identified in 15% of the patients [12] and may include a known history of hematologic conditions, local trauma, neoplasia, inflammatory conditions, structural anomalies, and medications. Further information is shown in Table 1 [10, 21, 22].

##### The role of antithrombotic therapy

More than 60% of patients hospitalized for severe epistaxis use antithrombotic agents. These medications are categorized into antiplatelets and anticoagulants [23, 24]. After analyzing every category, it becomes crucial to answer an essential question: should the antithrombotic medication be ceased or modified in the onset of a severe episode of epistaxis and which agent presents a safer option for treating a patient with epistaxis? Consulting cardiologists and hematologists is recommended before any decision is being made.

1. Antiplatelet medication

Antiplatelet drugs, such as aspirin and P2Y12 inhibitors (clopidogrel, prasugrel), are commonly used to prevent and treat arterial clot formation, thus resulting in primary and secondary prevention of cardiovascular disease (myocardial infarction and thromboembolic stroke) and are also administered as dual therapy in patients undergoing artery stenting [29]. As there is no antidote, during epistaxis, hemostasis is achieved by suspension of the antiplatelets, when possible, and performing any necessary treatment [30]. Due to their prolonged biological effect, stopping them is not a priority, and to address a critical uncontrolled bleeding situation, patients may require the transfusion of 5 units of platelets, whereas those on clopidogrel or prasugrel may necessitate the transfusion of ten units [33].

2. Anticoagulants

Anticoagulants target various parts of the coagulation process and they are primarily used to prevent and treat thromboembolic diseases (strokes caused by atrial fibrillation), thromboembolic pulmonary disease and clot formation induced by the presence of prosthetic heart valves [4, 29, 31]. Anticoagulants are categorized into parenteral and oral.

**Fig. 2:**
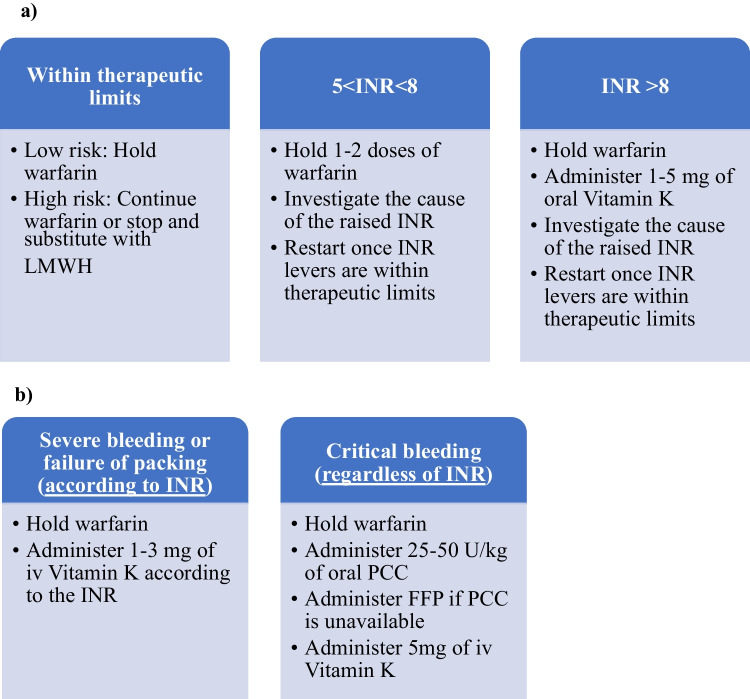
**a** Strategy for warfarin control: no active bleeding (regardless of packing). *LMWH* low-molecular-weight-heparin, *INR* international normalized ratio. **b** Strategy for warfarin control: active bleeding. *INR* international normalized ratio, *PCC* prothrombin complex concentrate, *FFP* fresh-frozen plasma

- Parenteral anticoagulants include unfractionated heparin, low-molecular-weight-heparin (LMWH), fondaparinux, and bivalirudin [31]. Out of these, LMWH is mostly used due to its superiority over unfractionated heparin and the smaller risk of bleeding. LMWHs have a short half-life (3–7 h) and are a great substitution for other anticoagulants in the case of surgery [25].
- Oral anticoagulants are classified into two main categories: Vitamin K antagonists, which include medications like warfarin and acenocoumarol, and Novel Oral Anticoagulants (NOACs) [31]. This chapter mainly focuses on warfarin, but the same principles apply to all Vitamin K antagonists.
  - Warfarin prolongs prothrombin time (PT), and thus, its therapeutic effect is monitored by measuring the International Normalized Ratio (INR) [31]. Bleeding typically occurs when the INR exceeds its therapeutic range [10, 31, 33]. It is often observed that patients with epistaxis are overly anticoagulated, with up to 79% of them having elevated INR levels at the time of presentation [10]. A multidisciplinary therapeutic approach between otolaryngologists, cardiologists, and hematologists is required. Patients on warfarin may be more prone to recurrence than patients on antiplatelets [18]. Figure 2 shows a suggestion of management.
  - NOACs target thrombin or factor Xa and have rapid onset of action and short half-time [31]. Bleeding under these medications is less severe than other antithrombotic agents [31]. In cases of intractable epistaxis, packing failure, or in the advent of surgery or embolization, suspension or substitution of NOACs by different agents should be advised after a multidisciplinary decision [24, 32].

### Initial assessment

Initial evaluation at the Emergency Room (ER) involves assessment of the grade of epistaxis using the Airway-Breathing-Circulation (ABC) approach, to secure the airway, ensure proper breathing, and stabilize the cardiovascular function [3]. In the case of a life-threatening episode inducing hemorrhagic shock, the top priority is to secure the airway by stopping the bleeding with posterior packing. Urgent BT may be necessary and patients need to be hospitalized for further evaluation and treatment [21].

Such episodes are rare; most cases present as serious or severe bleeding, not causing systemic symptoms. ABC evaluation and an early blood pressure measurement take place and anterior rhinoscopy reveals posterior epistaxis when a bleeding point cannot be identified. Other indications for posterior bleeding are bilateral bleeding, significant blood in the oropharynx, nausea, and hemoptysis or hematemesis [3, 21].

The initial approach is an attempt to identify the bleeding point using rigid nasal endoscopy, to cauterize it with silver nitrate or electrocoagulation [2, 5]. Complications may include septal perforation, infection, rhinorrhea, and increased bleeding [3]. Simultaneous bilateral cauterization may cause septal perforation and should be avoided [3, 34].

Failure of cauterization leads to nasal packing. Mild episodes are usually treated with anterior packing and resorbable materials may be used, especially in the case of coagulopathies or antithrombotic medication usage, to avoid mucosal destruction and recurrence [22, 34]. Non-resorbable materials used for anterior packing include Merocel, Rapid-Rhino, and ribbon gauze covered in paraffin [35].

Severe bleeding or failure of anterior packing leads to posterior non-resorbable packing (a double balloon catheter or a Foley catheter used with anterior paraffin gauze packing) [21, 34, 35]. Shargorodsky et al. revealed a higher success of these materials in bleeding suspension than chemical cauterization [36]. Patients need to be hospitalized for monitoring, comorbidity assessment, and potential further treatment [36].

Classification of epistaxis through history taking and physical, as well as laboratory, examination is important [3]. A crossmatch testing is imperative for potential BT. Imaging is not necessary, except in the case of an undiagnosed neoplasm causing recurrent episodes of bleeding [3].

### Nasal packing

Nasal packing is the treatment of choice for intractable posterior epistaxis when other simple measures or interventions, such as cauterization, have failed. Preparation of the nasal cavity and intravenous analgesia are imperative for the patient’s comfort. Packing in most cases is unilateral due to the lateral origin of bleeding [35]. Its availability, simplicity, and low cost make it easy to be found and used in most healthcare facilities. Despite its advantages, it should be mentioned that its failure rate is 25–60%, and it can be rather uncomfortable and painful to patients, obstruct breathing and cause local and systemic complications, which may occur in up to 68% of cases (Table 2**)** [1, 3, 4, 9, 21, 31, 34, 36, 37, 39]. The recommended time for the removal of the posterior packing varies in the literature, with suggested durations ranging from 24 h to 3 or 5 days. [3, 34, 35]. In the case of a double balloon catheter, gradual deflation is recommended after 24–48 h [3, 34]. In the event of packing failure, other second-line treatments, such as surgery or embolization, should be performed.

**Table 2:** Complications of nasal packing

Local	Systemic
Posterior dislocation of the pack in the airway	Aggravation of pre-existing sleep apnea
Reduced sense of smell	Allergic reaction
Increased hospitalization cost	Foreign body reaction
Sinusitis	Toxic shock syndrome
Synechiae	Hypoxia
Otitis media	Angina
Columellar/alar necrosis	Cardiac arrhythmia
Septal perforation	Sepsis
Facial edema	Infective endocarditis
Epiphora/dacryocystitis	Spondylodiscitis
Orbital cellulitis	Death
Cavernous sinus thrombosis	
Paraffinoma in case of paraffin gauze packing	
Misplacement of Foley catheter in cranial cavity in case of skull base fracture	

### Antibiotic prophylaxis

The utilization of systematic antibiotic prophylaxis in nasal packing remains a topic of debate in recent literature. Τhere is little evidence in recent studies for the administration of systematic antibiotics as prophylaxis, since there is an extremely low risk of potential nasal local infection, or Toxic Shock Syndrome (TSS), a potentially life-threatening condition [21, 40]. On the contrary, antibiotic usage may have adverse effects, such as increasing microbial resistance, clostridium difficile infection and Stevens-Johnson syndrome, as well as risk for anaphylaxis, which is higher than the risk of TSS [21, 40].

**Fig. 3:**
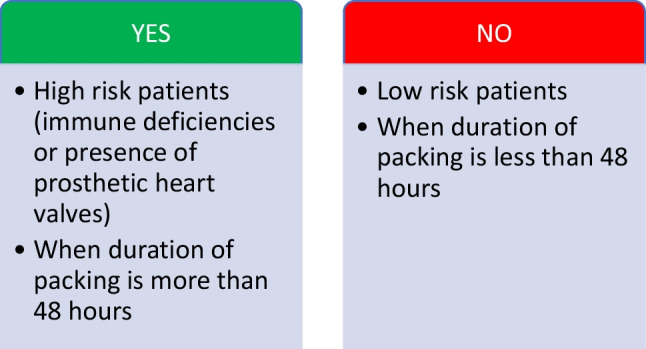
Suggested algorithm for antibiotic prophylaxis; to use or not to use?

Although there is strong evidence rejecting the usage of systematic antibiotics, large-scale studies have yet to be conducted. Low-risk patients who receive packing for less than 48 h do not seem to benefit from systematic prophylaxis. In certain cases, such as patients who require posterior packings for an extended period (more than 48 h) or individuals with immune deficiencies or prosthetic heart valves, the use of antibiotics should be strongly considered. These patient populations may be at a higher risk for developing rare complications, and the administration of antibiotics can help mitigate this risk [1, 21, 34]. All the above suggestions are summarized in Fig. 3.

However, despite the extremely rare incidence of few documented cases of TSS, clinicians are advised to use antibiotics according to their experience or follow these simple recommendations in accordance. The decision to administer antibiotics should be based on individual patient factors, clinical judgment, and the presence of specific risk factors that may increase the likelihood of infection.

### Definitive treatment

1. The first option is the use of posterior packing as a monotherapy and its removal after a period of 3–5 days, with potential concurrent antibiotic prophylaxis [34]. However, it is important to note that there is variation in the recommended duration of packing, with some authors suggesting removal after a maximum of 72 h. In the case of rebleeding during packing, it is possible to continue observation or perform a more effective re-packing [41]. In the case of re-packing failure or rebleeding after unpacking, patients should undergo surgery and/or arterial embolization. Minni et al. found a recurrence in 50% of cases, which can increase to 70% in patients with coagulation disorders [42]. There is no significant correlation in recurrence rates and the duration of nasal packing [36].
2. The second option is surgery, mainly involving the endoscopic ligation of the sphenopalatine artery (SPA) under general anesthesia. Other surgical approaches are the ligation of the anterior ethmoidal artery (AEA) concurrently or subsequently—in cases of recurrence—the internal maxillary artery (IMA) or the external carotid artery (ECA) [6, 43, 44].
  Endoscopic SPA ligation is a well-described procedure with success rates in controlling posterior epistaxis reaching up to 88–98% [6, 43]. Considering that the SPA may have 1–10 branches, it is advisable to look for accessory branches after the main trunk ligation by elevating the mucoperiosteal flap posteriorly to the turbinate, allowing for better visualization of the arterial anatomy [6]. Kitamura et al. revealed a 13.4% rebleeding rate after SPA surgery [44]. Postoperative short-term recurrence can occur due to the presence of anastomoses with other arteries or the recoiling of the SPA into the pterygopalatine fossa after unsuccessful cauterization [7]. Long-term recurrence may occur due to the revascularization of the SPA [8]. The potential complications of surgery can be minor, like nasal dryness crusting, acute sinusitis, intranasal adhesions, palatal numbness, or septal perforation, but they can also be more serious like rebleeding, decreased lacrimation, and inferior turbinate necrosis [9, 43, 44].
  AEA bleeding is less common and associated with previous surgery, head trauma, or may be spontaneous [9, 43]. AEA ligation may be performed concurrently with SPA ligation or subsequently in the case of embolization failure [37]. Surgical approaches are external via open, i.e., Lynch incision or endoscopic [43]. Possible complications are rebleeding, orbital injury, and skull base disruption [43].
  Posterior ethmoidal artery (PEA) ligation should rarely be considered, especially in patients under antithrombotic therapy, primarily due to its rare connection with severe epistaxis and secondary due to its proximity to the optic nerve [43].
  IMA ligation could infrequently be considered in the event of SPA ± AEA ligation failure before embolization [6, 44], although not recommended by the authors.
  ECA ligation requires neck surgery and should be reserved as a last resort in ongoing or recurrent bleeding after every treatment plan, including embolization, has failed or is unavailable [1, 44, 45].
3. The third option is endovascular embolization, routinely performed after surgery failure, and involves the embolization of the distal branches of the bilateral IMAs and the ipsilateral distal branches of the facial artery [8]. Success rates are similar as surgery, in 88–97% of cases [9]. Recurrence may appear due to failure to embolize the targeted vessels or bleeding from new sites [37]. Early rebleeding may be treated with further embolization [26]. Indications for primary usage of embolization include critical epistaxis with hemorrhagic shock or the inability for surgery in patients with poor cardiovascular status, as it can be performed under local anesthesia [8, 26]. Contraindications include severe carotid atherosclerosis, prior surgical ECA or IMA ligation, AEA bleeding, and certain anastomoses between the ECA and the internal carotid artery (ICA) [1, 8]. AEA embolization should be avoided due to the danger associated with the microcatheterization of the ophthalmic artery [8]. Complications occur in 2–17% of cases and are the direct result of the technique or by the accidental embolization of the facial, the ophthalmic, or the ICA. All possible complications are summarized in Table 3 and they are divided into minor and major [6, 9, 26, 37, 46].

**Table 3:** Complications of arterial embolization

Minor	Major
Bleeding recurrence	Facial skin necrosis
Temporofacial pain or numbness	Facial nerve paralysis
Headache	Monocular blindness/diplopia
Swelling	Hypoxia
Jaw claudication	Hypovolemia
Trismus	Stroke
Septal perforation	
Sinusitis	
Otitis media	
Groin hematoma

## Discussion

### Early endoscopic SPA ligation vs nasal packing

Posterior packing is the first line of management in intractable epistaxis and can be used alone as definitive treatment, whereas surgery can be reserved after failure of packing or rebleeding following packing removal [45]. Nevertheless, early SPA ligation—immediate surgery after initial assessment and packing—has been investigated in terms of cost-effectiveness, success, and complications rates [38, 41, 50].

Lakhani et al. proposed the Wexham Criteria for the early identification of patients in the need of SPA ligation (Table 4). They performed a retrospective analysis of 27 patients who underwent SPA ligation during a period of 8 years. Twenty-three of them had no recurrence 6 months post-operatively. The patients underwent emergency surgery if at least one of the criteria was fulfilled [27]. This leads us to the conclusion that in three out of four criteria—except for the one that requires three episodes of recurrent epistaxis and re-packing during a single admission—early SPA ligation is demanded, to avoid a recurrence [27]. In the case of an immediate packing failure, severe or critical bleeding upon arrival or at least three previous admissions for ipsilateral bleeding in the last 3 months, SPA ligation should be considered as an immediate definitive treatment after the initial assessment [27, 47].

**Table 4:** Wexham Criteria for SPA ligation

Persistent uncontrolled posterior epistaxis despite anterior or posterior packing
Three or more episodes of recurrent bleeding during a single admission requiring re-packing
Hemoglobin decrease greater than 4 g/dL and/or need for BT
More than three admissions for recurrent ipsilateral bleeding in the last 3 months

Early SPA ligation is more superior and cost-effective than packing, as it decreases the hospitalization time and, thus, the hospital cost per patients [38, 41, 50]. Dedhia et al. state the superiority of early surgery compared to prolonged packing, as it induces less pain and discomfort and causes fewer and less severe complications than packing, as well as less morbidity [38]. Although the brief packing for a period or 48 h may be more cost-effective than surgery, one should consider the recurrence rate of both practices [38]. Zou et al. also found lower recurrence rate when performing surgery than packing [47]—which in packing may reach up to 70%, especially in patients with coagulopathies [42]—resulting in a much higher success rate in bleeding suspension [13]. Kitamura et al. estimated the recurrence rate in SPA ligation to be 13.4% in their recent systematic review and meta-analysis [44]. In their study, a total of 896 cases of SPA ligation were assessed between 1955 and 2017. Additionally, they pointed out that out of the 33 selected studies, only 3 were conducted prospectively. This underscores the challenges associated with conducting this type of study in this medical condition, as previously mentioned [44].

The patients undergoing surgical intervention are generally younger than the patients receiving packing [13]. There are no significant differences between early or late SPA ligation in terms of age, sex, antithrombotic medication, anesthesiologist grade, the number of preoperative packings, or preoperative hemoglobin concentration [41]. However, Minni et al. suggest that patients with coagulopathies or under anticoagulant therapy with INR > 2 should wait until the INR decreases until surgery is performed [42].

Early SPA ligation should be avoided in medically unstable patients with a high anaesthesiologic risk. In these cases, prolonged packing, as well as potential embolization, should be considered as definitive treatments, despite the fact they may cause more severe complications than surgery [1, 13].

Surgery is also affected by the availability of operating rooms, the availability of materials and the treatment preferences according to the doctor’s experience [27, 41]. However, it should be considered as first-line definitive treatment, after initial assessment and temporary posterior packing placement due to its high success and low recurrence rate compared to prolonged packing [44, 48].

The direct comparison of early SPA ligation and packing is shown in Table 5**.**

**Table 5:** Direct comparison of surgery (early SPA ligation) vs. prolonged packing as definitive treatment

	Surgery	Packing
Success rate	Higher than 90%	Up to 70%
Recurrence rate	35.3%	50–70%
Complications	Up to 8,7%	Up to 68% (more severe)
Morbidity	Lower	Higher
Contraindications	High anesthesiologic risk Severe coagulopathies High INR	Patient suitable for surgery
Level of discomfort	Less discomfort	More discomfort
Hospitalization time	Shorter	Longer
Hospitalization cost	Lower	Higher (more than double)
Logistics	Medical expertise, materials	Medical expertise, materials
Comorbidities (preventing treatment)	General anesthesia inability Anticoagulation therapy	–
Grade (first-line treatment)	Serious/ Severe	Every grade

### Postoperative epistaxis

Early SPA ligation has been proven to have many advantages over packing [38]. Minni et al.’s study reveals that 20% of patients presenting with epistaxis had undergone nasal surgery, especially endoscopic sinus surgery [42]. Due to the high failure and complications rates of packing, early SPA ligation is highly recommended in cases of bleeding after sinonasal surgeries, to prevent recurrence, longer hospitalization times, and discomfort [44, 48].

### Ligation vs cauterization

Both ligating by clipping or cauterizing SPA are acceptable—although cauterization is more frequently used—and the decision is usually affected by the surgeon’s preference [7]. Rebleeding rate of cauterization is 7.2%, whereas ligation’s is 15.1%. Cauterization has higher complication rates at 10.2% instead of ligation at 6.4% [44]. The failure of cauterization occurs when the SPA has not been cauterized efficiently or when the artery recoils in the pterygopalatine fossa [35]. On the contrary, the failure of ligation is due to the slipping of the surgical clips or the failure to identify all branches of SPA [44]. Many articles reveal the superiority of cauterizing or cauterizing and clipping over solely clipping the SPA [7, 44].

### SPA ligation vs arterial embolization (Table 6)

**Table 6:** Direct comparison of surgery vs. arterial embolization

	Surgery	Embolization
Success rate	Higher than 90%	Higher than 90%
Recurrence rate	35.3%	13.4%
Complications	Mostly minor (8.7%)	Potentially major (2–17%)
Contraindications	High anesthesiologic risk Severe coagulopathies High INR	Severe carotid atherosclerosis Surgical ECA or IMA ligation AEA bleeding Anastomoses between ECA and ICA
Hospitalization cost	Lower	Higher (more than double)
Logistics	Medical expertise, materials	Medical expertise, materials
Comorbidities (preventing treatment)	General anesthesia inability Anticoagulation therapy	Severe carotid atherosclerosis
Grade (first-line treatment)	Serious/ Severe	Critical

Embolization can be used as an alternative monotherapy to either prolonged nasal packing or surgery, as well as a subsequent treatment in case of packing or surgery failure. Embolization can be used as an alternative to surgery in unstable patients to whom general anesthesia is contraindicated [37]. Embolization will be compared to surgery in terms of success and failure rates, complications rates, contraindications, hospitalization cost, availability of staff and materials, comorbidities, and severity.

#### Success

Success rates of both surgery and embolization are greater than 90% with mean success rates of 94.6% and 93%, respectively [48, 49]. Huyett et al. reviewed retrospectively 54 patients who underwent embolization for epistaxis between 2005 and 2015, and showed that embolization as a primary measure is effective at controlling epistaxis within the first 24 h following treatment with an immediate success rate of 92.6% [37].

#### Recurrence

The embolization rebleeding rate beyond 24 h is 35.3% according to Huyett et al. and the main reasons for the recurrence may be the failure to completely embolize the targeted vessels, the failure to embolize the correct arterial territory or the bleeding from a new site [37]. On the contrary, the rebleeding rate of SPA surgery is only 13.4% and the reasons are the imperfect clipping of cauterization failure [44].

#### Complications

Complications occur at 8.7% of SPA surgery cases as opposed to 2–17% in embolization [37, 44]. Although complications are less frequent in embolization, they tend to be more major [50]. Therefore, surgery is usually preferred as the first choice of treatment [46]. The mortality rate for both procedures is similar; however, embolization patients tend to have a higher risk for airway complications [49].

#### Contraindications

Contraindications of embolization have been discussed in a previous chapter [1, 8]. Surgery’s contraindications are the patient’s inability to undergo general anesthesia, severe coagulopathies, or high INR [42, 49].

#### Hospitalization cost

The hospitalization cost is a major factor, especially in small, district, low-budget hospitals. Many studies have concluded that the hospitalization cost associated with embolization is higher than surgery [46, 48]. The direct cost of embolization is up to 230% higher than surgery due to the more expensive materials being used [15]. The duration of hospitalization tends to be higher in patients who undergo embolization rather than surgery [49]. Leung et al. performed a risk analysis of treatment modalities in the literature published between 1980 and 2014. They assessed the cost-effectiveness of using surgery or embolization as a first-line treatment and concluded that SPA ligation should be performed prior to embolization [50].

#### Availability

The utilization of both procedures varies according to hospital characteristics, such as size, location, availability of necessary equipment and materials, and, most importantly, the availability of specialists who are comfortable of performing them [46, 48]. Embolization must be performed by an expert interventional radiologist, due to the major complication possibility [27, 44]. The learning curve in surgery may easily be achieved by otolaryngology specialists who perform nasal endoscopic surgery [42]. Therefore, the pursuit of either treatment is often inseparably dependable to logistics and, in certain cases, it may be the most important factor [46].

#### Comorbidities

Medically suppressed patients with a high anesthesiologic risk should proceed to embolization, because it can be performed under local anesthesia and/or sedation^31^. Patients who are on anticoagulation therapy or have a high INR are more likely to receive embolization, because it is less traumatic to the nasal mucosa [26, 42, 49].

#### Grade of bleeding

In the case of critical epistaxis, embolization could be performed as a first-line treatment, because the blood flow may be so significant, predisposing to surgery failure due to vision restriction and the potential need to intervene to more vessels [8, 26].

## Conclusion

There is a vast heterogeneity of results about epistaxis in the recent literature. Most cases, due to their emergency nature, are studied retrospectively and not prospectively. Large-scale double-blind studies are impossible and even unethical, limiting the research capabilities needed to better understand this entity. Most conclusions in this article are recommendations based on other studies and the guidelines proposed are informative for the better comprehension of the pathophysiology of epistaxis and the mechanics behind the decision-making.

## Abbreviations

ABC: Airway-breathing-circulation
AEA: Anterior ethmoidal artery
BT: Blood transfusion
CVD: Cardiovascular disease
CIMT: Carotid artery intima-media thickness
ECA: External carotid artery
ER: Emergency room
ICA: Internal carotid artery
IMA: Internal maxillary artery
INR: International normalized ratio
LDL: Low-density-lipoprotein
LMWH: Low-molecular-weight heparin
NOAC: Novel oral anticoagulant
PEA: Posterior ethmoidal artery
PT: Prothrombin time
SPA: Sphenopalatine artery
TSS: Toxic shock syndrome

## References

[1] Sireci F, Speciale R, Sorrentino R, Turri-Zanoni M, Nicolotti M, Canevari FR (2017). Nasal packing in sphenopalatine artery bleeding: therapeutic or harmful?. Eur Arch Otorhinolaryngol.

[2] Supriya M, Shakeel M, Veitch D, Ah-See KW (2010). Epistaxis: prospective evaluation of bleeding site and its impact on patient outcome. J Laryngol Otol.

[3] Beck R, Sorge M, Schneider A, Dietz A (2018). Current approaches to epistaxis treatment in primary and secondary care. Dtsch Arztebl Int.

[4] Spielmann PM, Barnes ML, White PS (2012). Controversies in the specialist management of adult epistaxis: an evidence-based review. Clin Otolaryngol.

[5] Thornton MA, Mahesh BN, Lang J (2005). Posterior epistaxis: identification of common bleeding sites. Laryngoscope.

[6] Sasindran V, John MS (2020). Role of endoscopic internal maxillary artery ligation in intractable idiopathic epistaxis. Indian J Otolaryngol Head Neck Surg..

[7] Dutta M, Haldar D (2017). Optimizing the outcome of transnasal endoscopic sphenopalatine artery ligation in managing refractory posterior epistaxis: a case-control analysis. Auris Nasus Larynx.

[8] Krajina A, Chrobok V (2014). Radiological diagnosis and management of epistaxis. Cardiovasc Intervent Radiol.

[9] Dubel GJ, Ahn SH, Soares GM (2013). Transcatheter embolization in the management of epistaxis. Semin Intervent Radiol.

[10] Melia L, McGarry GW (2011). Epistaxis: update on management. Curr Opin Otolaryngol Head Neck Surg.

[11] Ando Y, Iimura J, Arai S, Arai C, Komori M, Tsuyumu M, Hama T, Shigeta Y, Hatano A, Moriyama H (2014). Risk factors for recurrent epistaxis: importance of initial treatment. Auris Nasus Larynx.

[12] Kikidis D, Tsioufis K, Papanikolaou V, Zerva K, Hantzakos A (2014). Is epistaxis associated with arterial hypertension? A systematic review of the literature. Eur Arch Otorhinolaryngol.

[13] Zhou AH, Chung SY, Sylvester MJ, Zaki M, Svider PS, Hsueh WD, Baredes S, Eloy JA (2018). To pack or not to pack: inpatient management of epistaxis in the elderly. Am J Rhinol Allergy.

[14] Pope LE, Hobbs CG (2005). Epistaxis: an update on current management. Postgrad Med J.

[15] Min HJ, Kang H, Choi GJ, Kim KS (2017). Association between hypertension and epistaxis: systematic review and meta-analysis. Otolaryngol Head Neck Surg.

[16] Page C, Biet A, Liabeuf S, Strunski V, Fournier A (2011). Serious spontaneous epistaxis and hypertension in hospitalized patients. Eur Arch Otorhinolaryngol.

[17] Michel J, Prulière-Escabasse V, Bequignon E, Vérillaud B, Robard L, Crampette L, Malard O, SFORL Work-Group (2017). Guidelines of the French Society of Otorhinolaryngology (SFORL). Epistaxis and high blood pressure. Eur Ann Otorhinolaryngol Head Neck Dis.

[18] Khan M, Conroy K, Ubayasiri K, Constable J, Smith ME, Williams RJ, Kuhn I, Smith M, Philpott C (2017). Initial assessment in the management of adult epistaxis: systematic review. J Laryngol Otol.

[19] Elwany S, Ibrahim AA, Soliman AI, Bazak R, Ibrahim HA (2018). The significance of atherosclerosis in hypertensive patients with epistaxis. J Laryngol Otol.

[20] Kunz SM, Holzmann D, Soyka MB (2019). Association of epistaxis with atherosclerotic cardiovascular disease. Laryngoscope.

[21] Krulewitz NA, Fix ML (2019). Epistaxis. Emerg Med Clin N Am.

[22] Tunkel DE, Anne S, Payne SC, Ishman SL, Rosenfeld RM, Abramson PJ, Alikhaani JD, Benoit MM, Bercovitz RS, Brown MD, Chernobilsky B, Feldstein DA, Hackell JM, Holbrook EH, Holdsworth SM, Lin KW, Lind MM, Poetker DM, Riley CA, Schneider JS, Seidman MD, Vadlamudi V, Valdez TA, Nnacheta LC, Monjur TM (2020). Clinical practice guideline: nosebleed (epistaxis). Otolaryngol Head Neck Surg.

[23] Yaniv D, Zavdy O, Sapir E, Levi L, Soudry E (2021). The impact of traditional anticoagulants, novel anticoagulants, and antiplatelets on epistaxis. Laryngoscope.

[24] Buchberger AMS, Baumann A, Johnson F, Peters N, Piontek G, Storck K, Pickhard A (2018). The role of oral anticoagulants in epistaxis. Eur Arch Otorhinolaryngol.

[25] Biggs TC, Baruah P, Mainwaring J, Harries PG, Salib RJ (2013). Treatment algorithm for oral anticoagulant and antiplatelet therapy in epistaxis patients. J Laryngol Otol.

[26] Reyre A, Michel J, Santini L, Dessi P, Vidal V, Bartoli JM, Moulin G, Varoquaux A (2015). Epistaxis: the role of arterial embolization. Diagn Interv Imaging.

[27] Lakhani R, Syed I, Qureishi A, Bleach N (2013). The Wexham criteria: defining severe epistaxis to select patients requiring sphenopalatine artery ligation. Eur Arch Otorhinolaryngol.

[28] Marin E, Watelet JB, Gevaert P, Van Zele T (2019). Severe spontaneous epistaxis: retrospective study in a tertiary ENT centre. Eur Arch Otorhinolaryngol.

[29] Maier CL, Duncan A, Hill CE (2016). Pharmacogenetics in oral antithrombotic therapy. Clin Lab Med.

[30] Makris M, Van Veen JJ, Tait CR, Mumford AD, Laffan M, British Committee for Standards in Haematology (2013). Guideline on the management of bleeding in patients on antithrombotic agents. Br J Haematol.

[31] Shao C, Wang J, Tian J, Tang YD, Wang M (2020). Coronary artery disease: from mechanism to clinical practice. Coronary artery disease: therapeutics and drug discovery advances in experimental medicine and biology.

[32] Glikson E, Chavkin U, Madgar O, Sagiv D, Nakache G, Yakirevitch A, Wolf M, Alon EE (2019). Epistaxis in the setting of antithrombotic therapy: a comparison between factor Xa inhibitors, warfarin, and antiplatelet agents. Laryngoscope.

[33] Escabasse V, Bequignon E, Vérillaud B, Robard L, Michel J, Malard O, Crampette L, SFORL Work Group (2017). Guidelines of the French Society of Otorhinolaryngology (SFORL). Managing epistaxis under coagulation disorder due to antithrombotic therapy. Eur Ann Otorhinolaryngol Head Neck Dis.

[34] Bequignon E, Vérillaud B, Robard L, Michel J, Prulière Escabasse V, Crampette L, Malard O, SFORL work-group (2017). Guidelines of the French Society of Otorhinolaryngology (SFORL). First-line treatment of epistaxis in adults. Eur Ann Otorhinolaryngol Head Neck Dis.

[35] Randall DA (2006). Epistaxis packing. Practical pointers for nosebleed control. Postgrad Med.

[36] Shargorodsky J, Bleier BS, Holbrook EH, Cohen JM, Busaba N, Metson R, Gray ST (2013). Outcomes analysis in epistaxis management: development of a therapeutic algorithm. Otolaryngol Head Neck Surg.

[37] Huyett P, Jankowitz BT, Wang EW, Snyderman CH (2019). Endovascular embolization in the treatment of epistaxis. Otolaryngol Head Neck Surg.

[38] Dedhia RC, Desai SS, Smith KJ, Lee S, Schaitkin BM, Snyderman CH, Wang EW (2013). Cost-effectiveness of endoscopic sphenopalatine artery ligation versus nasal packing as first-line treatment for posterior epistaxis. Int Forum Allergy Rhinol.

[39] Pinto JA, Cintra PP, Sônego TB, Leal Cde F, Artico MS, Soares JS (2012). Severe complication of posterior nasal packing: case report. Int Arch Otorhinolaryngol.

[40] Hu L, Gordon SA, Swaminathan A, Wu T, Lebowitz R, Lieberman S (2021). Utilization of prophylactic antibiotics after nasal packing for epistaxis. J Emerg Med.

[41] McDermott AM, O'Cathain E, Carey BW, O'Sullivan P, Sheahan P (2016). Sphenopalatine artery ligation for epistaxis: factors influencing outcome and impact of timing of surgery. Otolaryngol Head Neck Surg.

[42] Minni A, Dragonetti A, Gera R, Barbaro M, Magliulo G, Filipo R (2010). Endoscopic management of recurrent epistaxis: the experience of two metropolitan hospitals in Italy. Acta Otolaryngol.

[43] Lin G, Bleier B (2016). Surgical management of severe epistaxis. Otolaryngol Clin N Am.

[44] Kitamura T, Takenaka Y, Takeda K, Oya R, Ashida N, Shimizu K, Takemura K, Yamamoto Y, Uno A (2019). Sphenopalatine artery surgery for refractory idiopathic epistaxis: systematic review and meta-analysis. Laryngoscope.

[45] İsmi O, Vayisoğlu Y, Özcan C, Görür K, Ünal M (2016). Endoscopic sphenopalatine artery ligation in posterior epistaxis: retrospective analysis of 30 patients. Turk Arch Otorhinolaryngol..

[46] Rudmik L, Leung R (2014). Cost-effectiveness analysis of endoscopic sphenopalatine artery ligation vs arterial embolization for intractable epistaxis. JAMA Otolaryngol Head Neck Surg.

[47] Zou Y, Deng YQ, Xiao CW, Kong YG, Xu Y, Tao ZZ, Chen SM (2015). Comparison of outcomes between endoscopic surgery and conventional nasal packing for epistaxis in the posterior fornix of the inferior nasal meatus. Pak J Med Sci..

[48] Lelegren M, Bhat K, Sheehan B, Lamichhane R, Han JK, Lam KK (2021). Variations in utilization and clinical outcomes for endoscopic sphenopalatine artery ligation and endovascular arterial embolization in a single multi-hospital network. Am J Otolaryngol.

[49] Sylvester MJ, Chung SY, Guinand LA, Govindan A, Baredes S, Eloy JA (2017). Arterial ligation versus embolization in epistaxis management: Counterintuitive national trends. Laryngoscope.

[50] Leung RM, Smith TL, Rudmik L (2015). Developing a laddered algorithm for the management of intractable epistaxis: a risk analysis. JAMA Otolaryngol Head Neck Surg.

